# Accurate In Vivo Bowman's Thickness Measurement Using Mirau Ultrahigh Axial Resolution Line Field Optical Coherence Tomography

**DOI:** 10.1167/tvst.11.8.6

**Published:** 2022-08-05

**Authors:** Samuel Lawman, Sharon Mason, Stephen B. Kaye, Yao-Chun Shen, Yalin Zheng

**Affiliations:** 1 University of Liverpool, Faculty of Science and Engineering, Department of Electrical Engineering and Electronics, Liverpool, UK; 2 University of Liverpool, Faculty of Health & Life Sciences, Department of Eye and Vision Science, Liverpool, UK

**Keywords:** ultra high resolution optical coherence tomography, Bowman's layer, epithelium, line-field, spectral domain, segmentation, thickness, keratoconus

## Abstract

**Purpose:**

The purpose of this study was to assess the accuracy, repeatability, and performance limits of in vivo Mirau ultrahigh axial resolution (UHR) line field spectral domain (LF-SD) optical coherence tomography (OCT) for the measurement of Bowman's and epithelial thickness, and to provide a reference range of these values for healthy corneas.

**Methods:**

Volunteers with no history and evidence of corneal disease were included in this study. An in vivo graph search image segmentation of the central cornea was obtained at the normal interface vector orientation. The Mirau-UHR-LF-SD-OCT system used has an axial resolution down to 2.4 µm in air (1.7 µm in tissue), with an A-scan speed of 204.8 kHz and a signal to noise ratio (sensitivity) of 69 (83) dB.

**Results:**

Nine volunteers were included, one of whom wore contact lenses. The repeatability of mean Bowman's and epithelial thicknesses were 0.3 and 1.0 µm, respectively. The measured 95% population range for healthy in vivo thickness was 13.7 to 19.6 µm for the Bowman's layer, and 41.9 to 61.8 µm for the epithelial layer.

**Conclusions:**

The measured thicknesses of Bowman's layer and the corneal epithelium using the Mirau-UHR-LF-SD-OCT were both accurate, with the range for healthy in vivo thicknesses matching prior confocal and OCT systems of varying axial resolutions, and repeatable, equaling the best value prior reported.

**Translational Relevance:**

T1. Development of a commercially viable clinical UHR OCT technology, enabling accurate measurement and interpretation of Bowman's and epithelial layer thickness in clinical practice.

## Introduction

Bowman's layer^1^ lies between the epithelium and stroma in some species, including human beings. It is acellular and composed of uniform (at optical scales) collagen. As a result, it generally displays less optical scattering than the epithelium and stroma. A primary histopathological feature^2^ of keratoconus is a breaking and disruption of Bowman's layer. Bowman's layer may also be affected in other acquired and developmental conditions of the cornea.^1^ Therefore, the measurement of its thickness, for use as a diagnosis and monitoring tool, is a topic of research interest^3^^–^^8^ and would be a valuable feature to measure in clinical practice. In addition, mapping of the corneal epithelial thickness, which is within the technical capabilities of current standard imaging techniques, including optical coherence tomography (OCT)^9^ and ultrasound,^10^ has become an additional diagnostic and monitoring tool for keratoconus and other conditions.

In vivo confocal microscopy (IVCM) is currently the clinical optical technique for the measurement of Bowman's layer thickness.^3^^,^^11^ The relative drawback of the technique is that it requires applanation of the patient's cornea. Although non-contact IVCM is possible,^12^ the resultant relative axial motion of the patient to the device makes accurate cross sections (thus thickness measurements) less feasible. The alternative approach is based upon OCT,^13^^,^^14^ which is a non-contact tomographic (3D) optical imaging technique using low coherence interferometry for the ranging in the axial direction. The 3D imaging can be achieved in either a full field (FF),^15^^,^^16^ a scanning point (SP; most common) or line field (LF)^17^^–^^19^ format. Standard resolution OCT systems, previously in time domain (TD)^13^ and currently in spectral domain (SD)^20^ and swept source (SS)^21^ forms, are widely used in ophthalmology.^22^^–^^24^

Currently, OCT is not generally used in clinics for quantitative measurements of Bowman's layer thickness. A reason for this is that the axial resolution of commercial clinical systems (range approximately from 5 to 20 µm in air, dependent on the system) is deemed insufficient (relative to the 13.7 to 19.6 µm total thickness of the layer) to define the boundaries accurately. A polarization sensitive (PS) OCT system published by Beer et al.,^25^ however, with a resolution in this range (8.7 µm in air), with conical scanning pattern and robust segmentation method gives remarkably repeatable (0.3 µm) measurements of Bowman's layer thickness. Given that the axial resolution of this method is a significant proportion (38% in tissue) of the average Bowman's thickness, validation of this measurement's accuracy with as high an axial resolution as possible is needed. Previous research systems with high axial resolution (HR; defined here as 5 to 3 µm in air)^5^ and ultrahigh axial resolution (UHR; <3 µm in air)^26^ have previously been used to measure the in vivo Bowman's layer thickness.

In this study, we present a new, relatively low cost, UHR LF SD OCT system using SC light source and Mirau configuration. A Mirau configuration has been developed in an LF TD OCT system,^27^ but the authors are not aware of it previously being published for an LF SD OCT system. Using in vivo image data of healthy corneas, we show with UHR axial resolution, optimum measurement direction normal vector orientation and robust semi-automatic segmentation over a reasonable length, that repeatability of Bowman's layer thickness measurement matches the best prior value in the identified literature. From the image and segmentation dataset produced by this study; we present a measurement of the 95% population range for the in vivo thickness of the central Bowman's (and epithelial) layers of healthy adult corneas. The accuracy of this technique is validated by comparing this range to independent prior publications.

## Methods

### LiveOCT (Mirau-UHR-LF-SD-OCT)

For UHR OCT, to achieve the theoretical axial resolution limit of a device, it is essential to minimize chromatic dispersion differences between the sample and reference arm of the interferometer. This is usually achieved by using a Linnik Interferometer^28^ including the predecessor^19^^,^^29^ to this device. Here, instead, we used a Mirau interferometer design, where the beam splitter and reference interface are in front of the objective lens, and share the same (inverted direction) optical axis as the sample arm. In optical profilometry/metrology, such interchangeable Mirau objectives are widely used,^30^ and can be swapped by the user to give the appropriate lateral resolution and image size for a given application.

**Figure 1.**
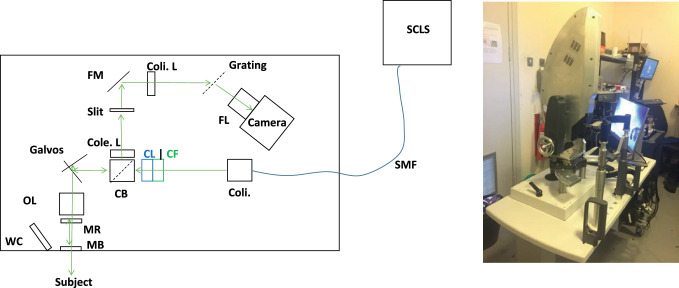
(Left) A schematic diagram of the Mirau UHR LF SD OCT system (LiveOCT) used in this study. SCLS, supercontinuum light source; SMF, single mode fiber; Coli., collimator; CF, custom filter; CL, cylindrical lens; CB, cube beam splitter; OL, objective lens; MR, Mirau reference; MB, Mirau beam splitter; WC, webcam; Cole. L, collection lens; FM, folding mirror; Coli. L, collimation lens; FL, final (camera) lens. (Right) Photograph of the complete LiveOCT system, as used.

Figure 1 (left) shows a schematic of the optics and a photograph of the UHR LF SD OCT (LiveOCT) device which was used for this study. The light source used was a Fianium Whitelase Micro with a 3 m length of S630HP single mode fiber output (NKT-Fianium Denmark/UK), collimated to a beam approximately 4 mm diameter (RC04APC-P01, Thorlabs). For further bandpass (700 to 1000 nm) filtering and to optimize effective spectral shape, a custom spectral filter was used (Laser 2000 Ltd., UK). A scan lens (LSM54-850, Thorlabs) was used as the objective lens.

The Mirau interferometer was constructed with a custom beam splitter (Laser2000 Ltd., UK) and a custom uncoated flat surface (Thorlabs) as the reference. Both components were made from fused silica, the reference plate was 1 mm thick, and the beam-splitter was 1.6 mm thick. These were arranged so that when the reference surface was aligned at focus, the focus at the subject was in the positive Fourier transform image and the dispersion mismatch was only 600 µm of fused silica.

The returned light is brought to focus by the collection lens (F = 75 mm achromatic doublet, AC254-075-B, Thorlabs) on the custom slit (10 µm × 10 mm, Thorlabs) of the custom imaging spectrograph. Unlike other slit-less designs^31^ of LF SD OCT, the slitted design^17^^,^^19^^,^^29^ was required here to gate out unwanted directly reflected light from the Mirau beam splitter, due to the relatively low numerical aperture (NA) of the objective lens. The spectrograph consisted of an F = 75 mm AC doublet (AC254-075-B, Thorlabs) collimation lens, a 600 l/mm holographic grating (Wastatch, US), an F = 75 mm camera lens (MVL75M1, Navitar via Thorlabs) was required to maintain image fidelity (focus) over the image of the spectrum and a Zyla 4.2P camlink (Andor, UK) camera.

For the results presented here, an integration time of 500 µs was used, which is slightly higher than the 311 µs identified by Nakamura et al.^32^ as ideal for their in vivo LF SD OCT system. For the data collected by trainee users (*N* = 5), the number of frames discarded (i.e. negligible image signal remaining after washout) by the described automated thresholding algorithm (see the Correlation Averaging and Segmentation section) varied between 0 and 80%, with a median of 6%. For an experienced user for all captures (*N* = 6) on 1 patient, only 1 frame out of the 300 total was discarded. It should be noted that the thickness repeatability reported by this paper was taken from the trainee captured data, therefore, we concluded that the 500 µs integration time used was acceptable.

Figure 1 (right) is a photograph of the developed setup. The optical enclosure was mounted on a mechanized mount with 5 degrees of freedom, and a motorized lifting ophthalmic table. A custom, extra wide, and stiff, designed head and chin rest, with separate handles, were constructed to keep the subject stable and comfortable. The unit included a workstation PC (HP Z4 G4, Intel Xeon W2123 3.6 GHz 4 cores, 32 GB RAM, NVIDIA Quadro P400), for live OCT display, data capture, and previewing. The software was custom written using C++, C++/CLI, C#, SQL, and Winforms, in Microsoft Visual Studio 2013.

The SD OCT image reconstruction followed the same process described in Ref. 19. In short, after the capture of the raw spectra images, an averaged blank (reference path signal only) image was subtracted. The spectra were then resampled to equal frequency space and a pre-calculated digital spectral shaping window image were applied. For the signal to noise ratio (SNR; sensitivity) analysis and going into a future clinical study, a fixed (premeasured using a glass interface and Hilbert transform analysis to compensate instrument dispersion only) complex numerical dispersion correction (NDC) array was multiplied. For the final usability study (image data presented), for convenient symmetry of folded (beyond Δz = 0) images, this was not applied. The degradation of axial resolution (and SNR) without NDC was 8%, which was not significant. For the anterior of the cornea, sample dispersion is unlikely to be a significant detriment to the produced images. Fast Fourier transform was then performed and the log base 10 of the amplitude (α dB) was presented as the image.

The light emissions at the location of the subject's eye (near field) were within IEC 60825-1:2014 class 1 limits. The average angular subtense (eye focus at infinity) of illumination was 23.85 mrad, C6 = 15.9, T2 = 30.36, C7 = 1, C4 = 1 (700 nm), giving a class 1 total power limit of 5.5 mW. The power output from the device was 3 mW which is within this limit.

### Interface Normal Vector Imaging Orientation

There are two processes that can distort axial length measurements in optical methods. First, is the group velocity delay, which is taken as basic knowledge for all OCT and other LCI systems. The second cause is refraction of light due to change in phase refractive index at material boundary. This, as will be shown for IVCM in the Literature Consensus of Healthy Bowman's Layer Thickness section, is more often overlooked. For OCT, however, axial ranging is not achieved by focus, so refraction is not an issue when all interfaces’ normal vectors closely corelates to the incident light propagation direction (normal interfaces). Non-normal interfaces will refract the light path, meaning that the signal from underneath is not coming from where it appears. This distortion can be corrected,^33^ but this introduces extra complexity to the method and sources of error.

To overcome refraction distortion effects, the imaging was done at the normal vector orientation to the corneal surface and layer interfaces. Even more significant than making refraction error negligible, is at this orientation, the specular reflections from the layer interfaces are the most strongly captured features in the images. This makes image segmentation of the boundary straight-forward and removes any systematic interpretive ambiguity of real interface position, which occurs if a segmentation has to define a boundary between two volume scattering materials. The orientation of imaging is a key factor in why the repeatability of Bowman's and epithelial thickness in this work is better than most previous reports. Note that although this study looked at only the center, the normal orientation can be achieved for any part of the cornea with appropriate fixation target positioning.

### Test Subjects

Research ethics approval for the development in vivo usability testing of the device, including the final usability study results presented here, was granted by the University of Liverpool's Health and Life Sciences Research Ethics Committee (Ref. 4289).

Nine adults acted as test subjects, as well as acting as test users. All these persons had no known condition that would adversely affect the cornea. No statistics regarding age or gender were taken for this final usability study.

### Correlation Averaging and Segmentation

**Figure 2.**
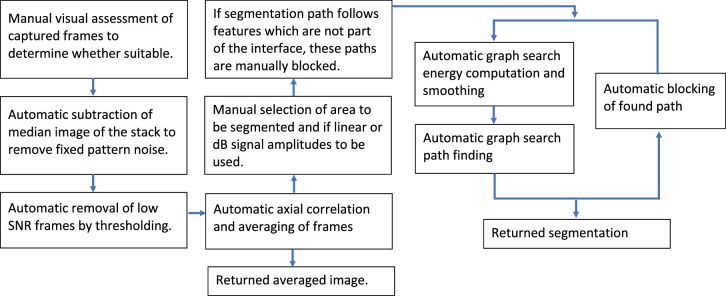
Flow chart of correlated image averaging and image segmentation method.

A 2D capture consisted of (a stack of) 50 consecutive images (frames, total capture time = 0.125 seconds). The flow chart for the post processing is shown in Figure 2. In the first stage, the reconstructed OCT image data was assessed to check that the cornea was in the correct position in all 50 frames (i.e. the front of the cornea top and center and not inverted). If the stack was accepted, the median image was taken and subtracted from each frame (the same principle as Ref. 34 but in 2D rather than 1D) to remove fixed pattern artifacts. To identify and remove any frames with negligible image contrast, thresholding to a minimum pixel value and minimum pixel per frame count was used. To compensate for axial motion between frames, an area covering the position of the strongest and desired image features (in this case, it would cover the anterior cornea surface and Bowman's layer interfaces) in all the frames was manually selected. For this area, the remaining frames were cross-corelated to the frame with the highest threshold pixel count using a relatively simple 1D (axial dimension only) nearest pixel algorithm. With the frames all lined up, a mean of linear amplitudes image was taken. The area where the three interfaces of interest were clearly visible was selected. The graph search segmentation method described in Ref. 35 based on code from Ref. 36 was used. Due to the signal from the interfaces dominating internal scattering signal, only the amplitude (not differential) energy function was required. The image and energy function smoothing were minimal at 2 × 2 (median filter) and 2 × 2 (top hat convolution) pixels, respectively. Three path searches were then returned. If necessary, incorrect paths were manually blocked before repeating the segmentation method.

## Results

### LiveOCT System Performance Values

Table 1 gives the performance measurements of the LiveOCT system as used in the final usability study.

**Table 1.** The Measured Performance Values of the Developed Mirau UHR LF SD OCT System Used in This Study

	Measured Value
Image (A-scan) depth	1.23 n_G_.mm
B-scan length	2.29 mm
Axial resolution	Without NDC 2.6 n_G_.µm, with NDC 2.4 n_G_.µm
Lateral resolution	∼ 20 µm
Axial image rate	204.8 k A-Scans/s
Single frame sensitivity	83 dB
Single frame dynamic range (Glass interface signal/empty standard deviation)	69 dB

**Figure 3.**
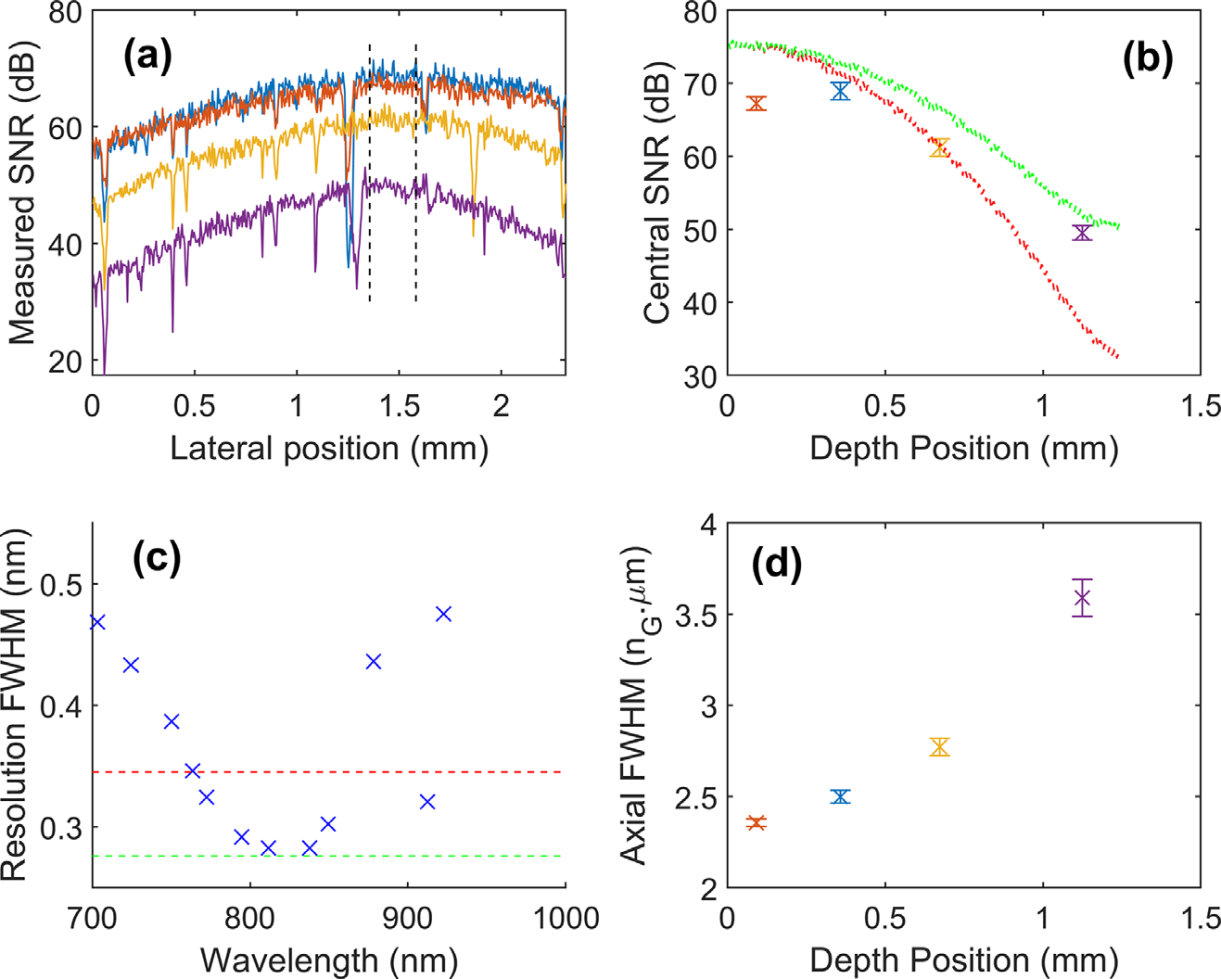
(**A**) Measured SNR across the B-scan image at different image depths (see **B** or **D** for depths). Between the *black dashed lines*, (**B**) and (**D**) show the mean (x) and standard deviation (error bars), of the SNR and axial resolution respectively, as a function of depth position. (**C**) Measured spectrograph resolutions. *Dotted green* and *red lines* show 2.0 and 2.5-pixel resolutions, respectively, the resultant modelled roll off curve for these resolutions are also shown in **B**.

The SNR and roll off for the system were measured, and then compared to a numerical model of photo-electron shot noise and spectrograph resolution on simulated ideal signals. Figure 3a shows the measured LF lateral SNR fall off, which can be reduced by use of a Powell lens.^37^ Figure 3b shows the measured axial SNR roll-off curves with modeled values for two different spectrograph resolutions. Figure 3c shows how these spectrometer resolutions used in the models compare to the measured values across the spectrum. The nonuniform spectral resolution (increasingly nonuniform washout of signal with depth) was a cause of the axial resolution roll-off shown in Figure 3d.

### Bowman's and Epithelial Layer Segmentation

**Figure 4.**
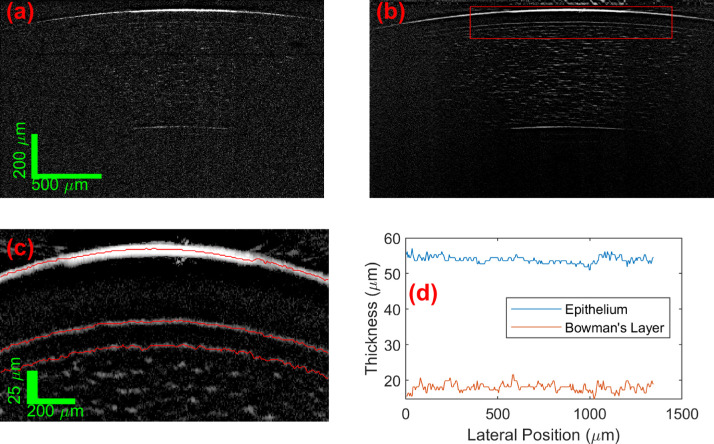
(**A**) Single LiveOCT frame (after fixed pattern noise removal) and (**B**) automate correlated average of the 50 capture frames of an in vivo cornea. The *red box* is the area zoom in on (**C**), with the *red lines* showing the automatically segmented interfaces. (**D**) Is the resultant measured thickness profiles for the epithelial and Bowman's layers.

Figure 4 shows an example of one of the 2D captures, and the segmentations of Bowman's and epithelial layer thicknesses. In the raw (after subtraction of fixed pattern noise) unaveraged frames, the signal from the front and rear interfaces of the cornea are visible, along with the Bowman's layer interfaces and individual scattering centers within the stroma (these are likely to be keratocytes and/or lamella interfaces). The correlated-average image significantly increases the contrast of the features above the noise in the image. Zooming in on the central part of the epithelial and Bowman's layers, the reflection from the interfaces of these are clearly resolved by UHR LF SD OCT at the normal orientation, making it ideal for accurate measurement of the thickness of these layers. The device is capable of imaging the cornea at any location by control of the location of the external fixation target of the patient's gaze (and also motion axis of freedom of the device if required), which would be important for the best keratoconus diagnostic metrics.^5^ For the purpose of this study, to measure technique repeatability and define healthy central corneal values, a constant lateral position was used. With strong interface signals, the graph search segmentation based on the (nondifferentiated) image amplitude was straight-forward to automatically segment the interface locations, and obtain layer thickness (using n_G_ = 1.387 following^38^ and closely matching^39^). The air – epithelium (tear film, see below), and epithelial – Bowman's layer boundaries are smooth well-defined boundaries, whereas the Bowman's layer – stroma interface measurement output has more apparent roughness, which is likely partially due to the existence of the significant signals from inside of the cellular stromal layer (Bowman's layer is acellular^1^). This will not be distinguished from the interface signal if adjacent. The standard deviations of the measured Bowman's layer thickness profiles were between 1 and 2.5 µm, which is significantly lower than the axial imaging resolution of current commercial ophthalmological OCT systems and covers the axial imaging resolution (in tissue) of the UHR LF SD OCT device itself, so this increase in measured interface roughness is not a significant issue.

**Figure 5.**
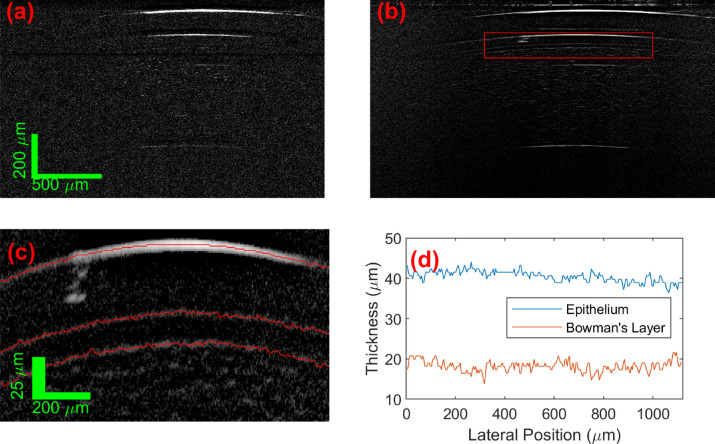
(**A**) Single LiveOCT frame (after fixed pattern noise removal) and (**B**) automate correlated average of the 50 capture frames of an in vivo cornea with contact lens. The *red box* is area zoom in on (**C**), with the *red lines* showing the automatically segmented interfaces. (**D**) Is the resultant measured thickness profiles for the epithelial and Bowman's layers.

One of the test subjects was wearing contact lenses (Fig. 5). Both the front and rear interfaces of the contact lenses were visible along with the interfaces visible in subjects without a contact lens. The only notable feature of this example was the low epithelial thickness. This was a real difference in epithelium thickness and not a measurement error. At least one previous study^40^ has documented and characterized epithelial thinning due to wearing contact lenses. The measured Bowman's thickness was within the range of the non-contact wearers.

**Figure 6.**
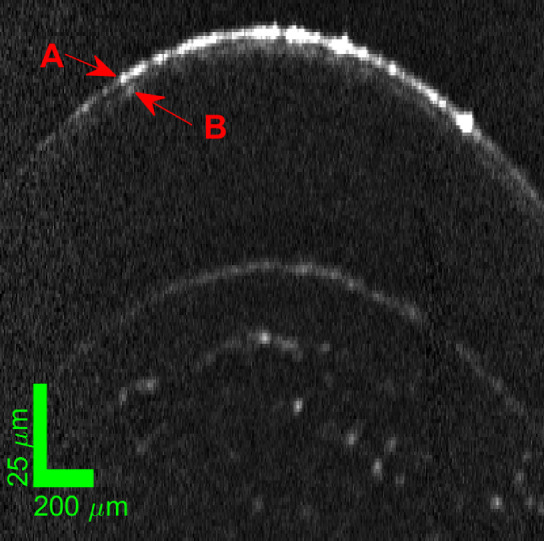
An example LiveOCT image where a thick tear film is apparent. (**A**) Shows the location of apparent air – tear film interface. (**B**) Shows the location of apparent tear film - epithelium interface. This tear film corresponded with an increase in the combined epithelium + tear film thickness measured.

In the above examples, the tear film was not visually resolvable whereas in Figure 6 the interfaces of the tear film were visible. There are two reasons why the tear film is resolvable in this case. First, it is deduced that this was taken after the subject blinked. A capture taken immediately prior (to the blinking) did not show the tear film and gave a 2 µm reduction in epithelial thickness. As the amplitude of Fresnel reflection from the tear film – air interface is much greater than the scattering from the epithelium – tear film interface, all presented segmentations of the epithelial layer includes the tear film. The tear film is a dynamic layer, with variation of thickness of 2 µm in a blink cycle being well within expectations.^41^ The second reason for resolution of tear film in Figure 6 is apparent from comparison of the image with the corneal surface in Figure 4. Compared to Figure 4, the signal from the air-tear film interface in Figure 6 was much weaker, with the PSF sides not extending as far due to the reduced amplitude. In Figure 4, even if the tear film was the same thickness, the signal from it would be masked by the much bigger air-tear film interface reflection. The reason for this difference between Figures 4 and 6, will be a lateral shift of imaging location away from the normal orientation position in the confocally gated dimension. Although the air-tear film signal is specular, and its magnitude highly dependent on position, the signal from the mucous tear film-epithelium boundary was clearly diffuse (see Fig. 6), so its amplitude remains unchanged with slight lateral position changes. The geometrical distortion of measured thickness due to this amount of mispositioning from the normal orientation was negligible in comparison to the measured differences, therefore, the reason for the epithelium thickness fluctuations reflects changes in the tear film thickness. A larger repeatability error for the thickness of the epithelium, compared with Bowman's layer, has been consistently reported,^3^^,^^8^^,^^25^^,^^42^ with tear film variability likely to be a main cause.

### Produced Bowman's and Epithelial Layer Thickness Dataset for Healthy Corneas

**Figure 7.**
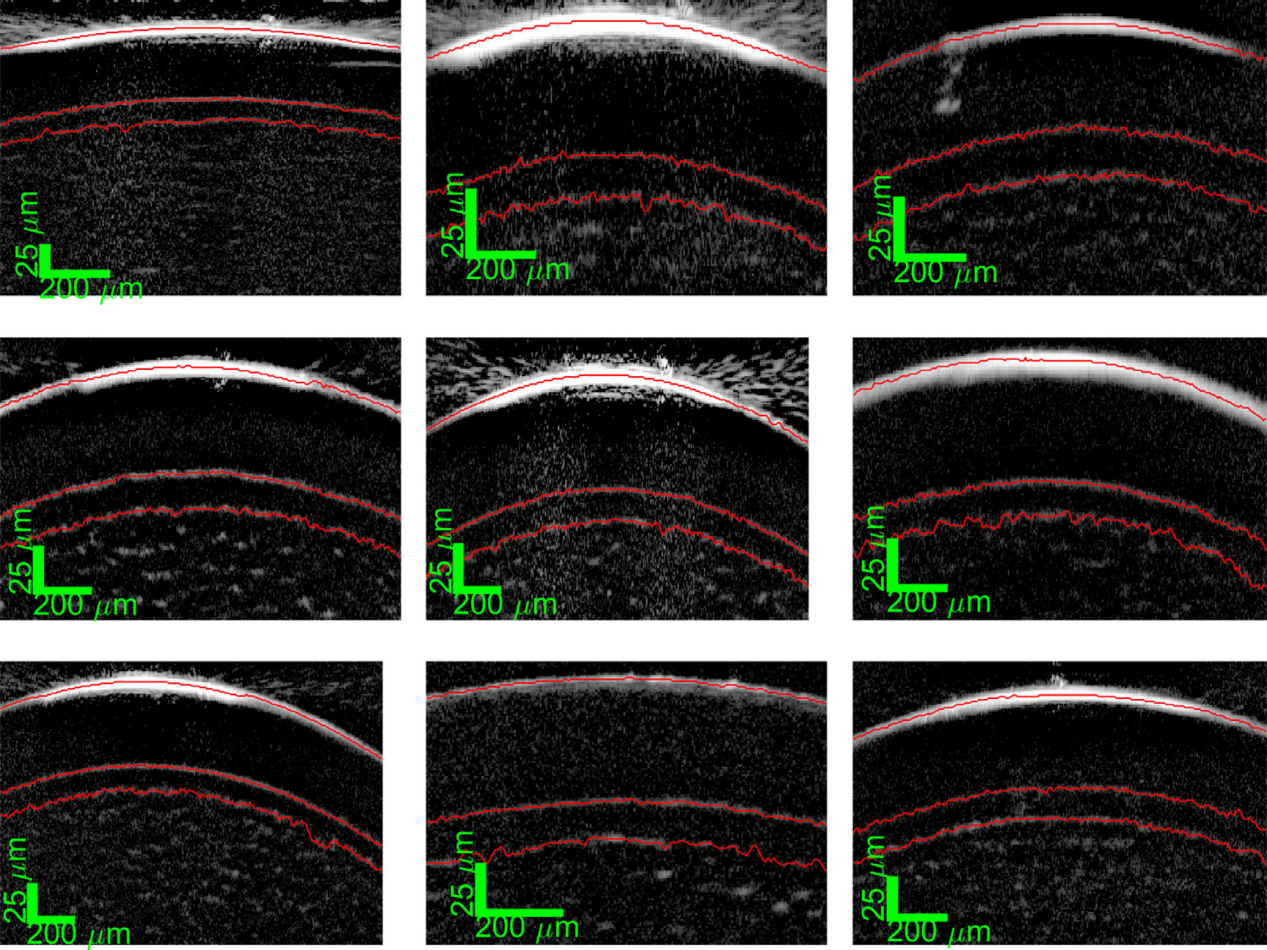
Example segmentations from all nine healthy test subjects.

Figure 7 shows an example for each of the 9 eyes. These show consistent image and segmentation quality. The measurements of these nine healthy eyes provided a reasonable dataset for the expected in vivo epithelial and Bowman's layer thickness.

**Figure 8.**
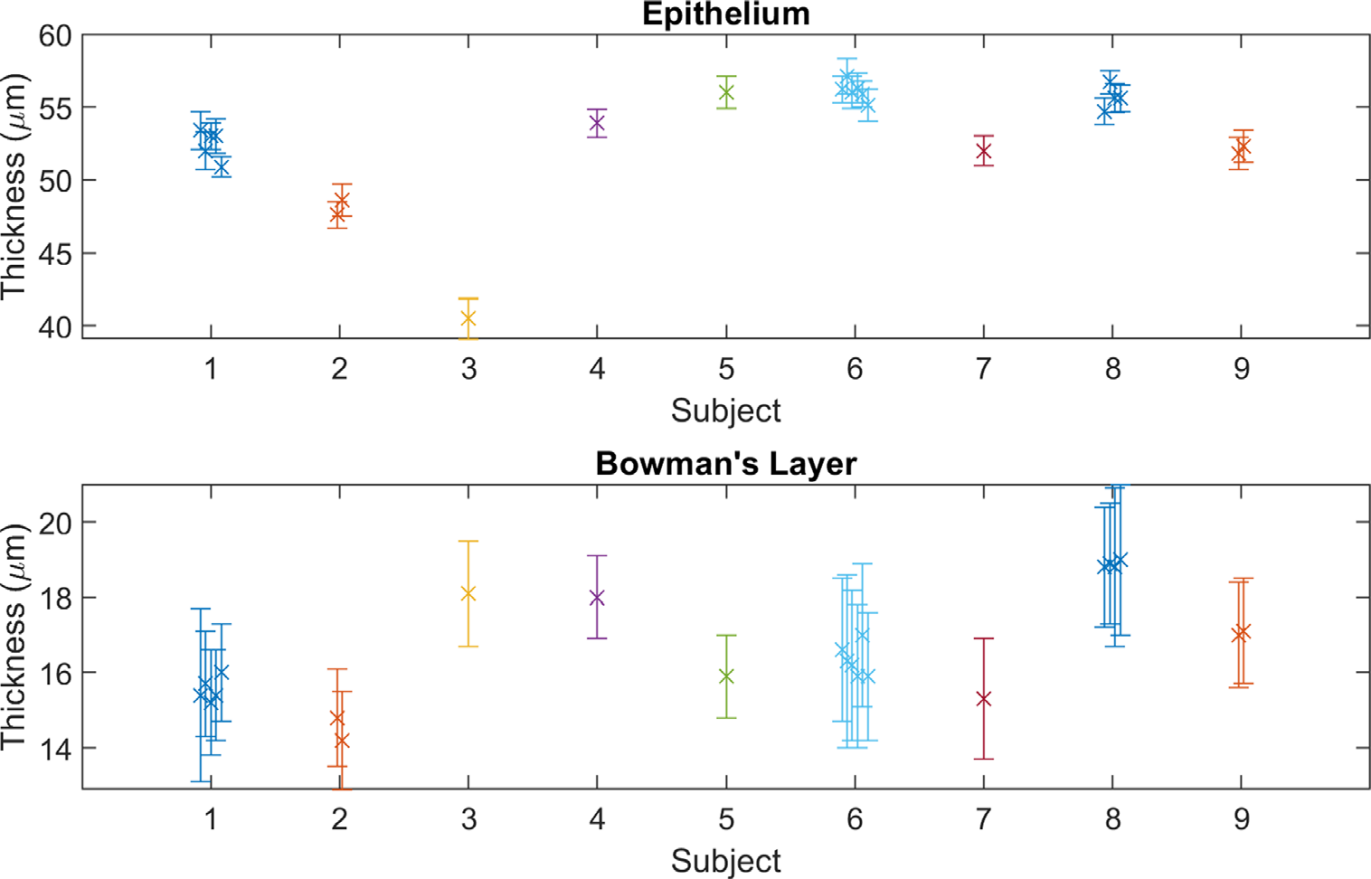
Mean ± standard deviation of all measured thickness profiles of the epithelial (*top*) and Bowman's (*bottom*) layers.

Figure 8 shows the measurements of epithelial and Bowman's layer thickness of all 9 test subjects. As discussed above for epithelial thickness, the subject wearing contact lenses (subject 3) was an outlier (40.5 µm), whereas the rest of the test subjects had a measured 95% population (2 times the standard deviation, assumed normal distribution) range of 41.9 to 61.8 µm. The measured 95% population range of thickness of Bowman's layer was 13.7 to 19.6 µm. Excluding the contact lens wearer, the correlation between epithelial and Bowman's thickness is moderate, with a Pearson coefficient of 0.6.

**Figure 9.**
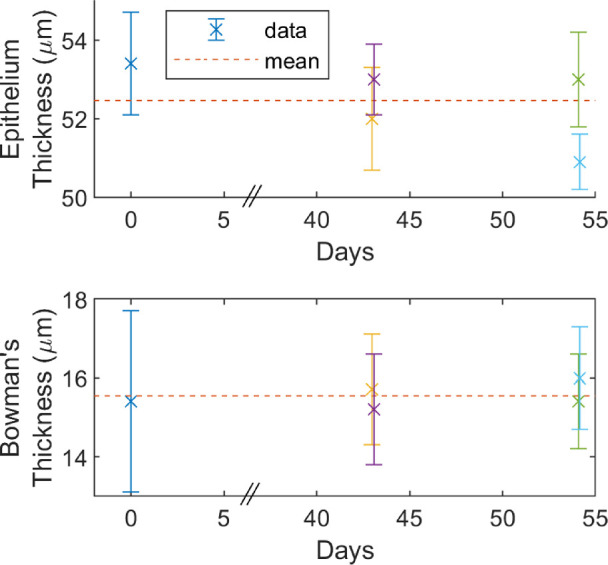
For a test subject measured at multiple different dates, by different operatives, the mean ± standard deviation of the epithelial (*top*) and Bowman's layer (*bottom*) profile thicknesses. Each operator is represented by a different color. The *red dotted lines* are visual aids, to demonstrate lack of trend, showing the mean value of the means.

The repeatability of the mean thickness measurement is shown in Figure 9. These thicknesses, for one test subject, were measured from images taken over a period of 55 days by multiple test operators. No trend with time is detected, with a standard deviation between thickness profile means of 1.0 and 0.3 µm for the epithelial and Bowman's layers, respectively. Given the lack of apparent trends for epithelial thickness in time or space (i.e. in the thickness profiles), it is likely the increased variation in its thickness for the epithelium was due to variation in the tear film thickness between measurements. Note that the tear film does not impact the repeatability of Bowman's layer thickness measurements, which does not share an interface with it. Alternatively, taking the mean of the standard deviation of mean profile values for all test subjects with multiple measurements, the repeatability values were 0.7 and 0.3 µm for the epithelial and Bowman's layers, respectively.

## Discussion

### LiveOCT

The ultra-high axial resolution (2.6 and 2.4 n_G_.µm [1.9 and 1.7 µm in corneal tissue] without and with numerical dispersion correction, respectively) of the LiveOCT system is much better than current commercial clinical ophthalmological OCT systems on the market. This axial resolution is similar to our previous device where a demonstration of the comparative differences of the imaging, compared to current commercial clinical OCT systems, has been made.^29^ In this study, we have used the improved ability to resolve the thinner layers of the cornea for the accurate assessment of Bowman's layer thickness. Here, we reiterate, to the authors knowledge, no current commercial system currently claims to segment the Bowman's layer. However, with this device and methodology we found resolving and segmenting the Bowman's layer to be relatively easy. In addition, the LiveOCT device is not impacted by in situ contact lenses for these measurements.

However, further applications of this UHR ability to other layers, such as tear film^41^ and Descemet's membrane,^43^ are also possible, and will be the subject of future work. In addition, we note that individual keratocyte scattering centers are resolved within LiveOCT images. Using the device for measurement of keratocyte density is also a possibility.

LiveOCT's raw imaging speed of 204.8 kA-Scans/second is competitive with the fastest new and prospective commercial clinical devices currently on the market. However, this rate is not yet at a fundamental limit for LF-SD-OCT (i.e. by the integration time and optical properties of the setup) but by the technical constraint of the imaging speed of the camera. The replacement of the camera with a high speed one, such as used in Ref. 44, approaching a duty cycle of 1 would mean in vivo MHz rates are feasible with this technology.

There are two main areas where further improvements to the technology can be made. First, improvements to the custom spectrograph optical design, to give a flatter and lower spectral resolution curve, are potentially feasible and would reduce both SNR and axial resolution roll off. Second, a higher image SNR and absolute sensitivity is often desirable. A larger pixel size, giving higher electron well depth, camera would provide a higher theoretical single frame shot noise SNR limit. However, increasing integration time to collect these extra photons is not practical due to washout, although there is some room to improve optical efficiency through the device. Instead, future faster camera electronics increasing the duty cycle close to 1 would mean a frame rate of 2000 frames/second (1.0 M A-scans/second). The output frame rate can then be traded for sensitivity by the averaging of raw frames, the expected return would theoretically be a 10 dB increase for every power of 10 frames averaged. Modern computing power (particularly via massively multithreaded GPU calculation) should allow the real-time correlated averaging of multiple frames.

In this manuscript, we have demonstrated that the (automatic) graph search path is reliable as the Bowman's layer boundaries. Minimizing the manual input required for the overall segmentation process is not a novel and insurmountable problem, and will be an area of future development.

### Clinical Lateral Position Repeatability

One theoretical limitation that may impact the repeatability of Bowman's (and epithelial) thickness measurement, in a translated clinical setting, is inconsistent lateral positioning between measurements of different visits months, or even years, apart. In the case that there are no changes to the cornea, using a standard positioning setup of the device, a set fixation target position and imaging at the normal vector orientation (a product of the standard alignment instructions for use) means the lateral measurement position is highly repeatable. This is evidenced by the excellent repeatability results of this study. It can be concluded for the method presented, inter-visit lateral mispositioning is not a significant source of measurement error for healthy or non-changing corneas. In the case where there has been a macroscopic asymmetric change in corneal shape, such as in keratoconus progression, and assuming a scenario where measurement of this change has not been undertaken, a lateral shift between measurement locations would be expected. Any change measured, therefore, may be the result of direct change to the Bowman's thickness or lateral shift of the measurement, but in either case would be a successful detection of change. To help discriminate, for every acquisition, the “webcam” (low cost USB endoscope camera) in the device captures an image of the iris and pupil. With calibration, this will allow identification of the lateral location on the cornea of any data capture with high precision.

### Literature Consensus of Healthy Bowman's Layer Thickness

One research thread that produces outlier results for the in vivo thickness of the Bowman's layer is the method of Germundsson et al.,^4^ using a commercial device (HRT3-RCM, Heidelberg). They reported mean Bowman's layer thickness for healthy eyes for 2 variants of their method, which was 13.2 and 9.1 µm, significantly lower than other in vivo studies. This could be caused by their assumption that their axial scaling is not dependent on refractive index and that they did not account for tissue volume change^45^ when using histology as a gold standard for comparison. This could be caused by their assumption that their axial scaling is not dependent on refractive index and they did not account for tissue volume change^45^ when using histology as a gold standard for comparison. If we apply the normal interface paraxial (small NA) approximation correction:

$$\begin{array}{c} Z_{r}=nZ_{a}, \end{array}$$

where Za is the apparent (reported) thickness and n is the phase refractive index at the wavelength of light used (approximately 1.376 for the cornea, visible light), the reported values become 18.2 and 12.5 µm, which are much closer to other reported values. Previous^3^ IVCM work has discussed a calibration relationship for real depth provided by the manufacturer, and this work produced a mean thickness value (16.6 µm) matching the values reported with OCT (see below).

Table 2 shows the reported^3^^,^^5^^–^^8^^,^^25^^,^^42^^,^^46^^–^^51^ in vivo thickness ranges of the Bowman's layer, measured by SR (PS) HR and UHR OCT, IVCM and this paper's UHR OCT. We have not included work based on Ref. 4 due to the identified potential issues and its discrepancy with other reported values. The mean value of the reported mean values is 17.1 µm and for the reported ranges (95% population = 2 times the standard deviation [assume normal statistical distribution]) is 13.5 to 20.7 µm. From this study, the mean value was 16.6 µm and the 95% population range of 13.7 to 19.6 µm. Given that the standard deviation of discrepancy of the mean value between previous studies is 1.1 µm, this study sits right in the middle of the literature consensus. Neither have we identified any reasons there may be significant systematic errors with any of these values, and therefore conclude that both this cited literature range and the range produced by this study are accurate.

**Table 2.** Trusted Literature Values for the In Vivo Thickness Range of Healthy Human Bowman's Layers and Reported Thickness Value Repeatability of the Techniques Used

Method (Axial Resolution in Air [µm])	Paper	Reported Population Mean and Standard Deviation or Range (µm)	Repeatability (Standard Deviation or Difference Between Independent Measurements)
IVCM (9)	Li et al. 1997^3^	16.6 ± 1.1	2.3
SR (PS) OCT (8.7)	Beer et al. 2018^25^ and Pircher et al. 2020^46^	16 ± 2	0.3
HR OCT (4.2)	Shousha et al. 2014^5^	15 ± 1	–
HR OCT (4.2)	Eleiwa et al. 2020^47^	14 to 21	1.1
HR OCT (4.2)	Hu et al. 2021^6^	17.5 ± 2.0	–
HR OCT (4.2)	Li et al. 2021^7^	18.0 ± 1.6	–
HR OCT (4.2)	Tao et al. 2011^48^ and Lian et al. 2013^49^	17.7 ± 1.6	–
HR OCT (4.2)	Xu et al. 2016^8^	–	1.3
HR OCT (4.2)	Xu et al. 2015^42^	–	0.5
UHR OCT (1.8)	Schmoll et al. 2012^50^	18.7 ± 2.5	–
UHR OCT (1.5)	Yadav et al. 2012^51^	16.7 ± 2.6	–
UHR OCT (2.4)	This paper	16.6 ± 1.5	0.3

Table 2 also shows the reported or derived repeatability values of the Bowman's layer thickness measurements. This study has the joint best repeatability value, along with the SR PS OCT system developed by Beer et al.,^25^ at 0.3 µm. The two significant common factors explaining why this study and Beer et al.^25^ produced the best repeatability values are a robust segmentation method giving a consistent mean thickness over a segmented length, and imaging at near normal orientation to corneal interfaces, this being achieved by Beer et al.^25^ over the whole cornea by their conical scanning method. For HR and UHR OCT systems, our value is better than the previous range of 1.3 to 0.5 µm.

### Lower Cost UHR OCT Discussion

Despite demonstrated^26^^,^^52^^,^^53^ clinical benefits of anterior segment UHR OCT, the authors are not aware of any such commercial clinical devices currently on or earmarked to come onto the market. One reason for this is the lack of an affordable suitable broadband light source. Reported UHR OCT systems have typically made use of femtosecond lasers^26^^,^^52^ and high specification supercontinuum (SC) light sources,^53^ with retail prices similar to that of current complete clinical OCT systems. For a lower cost light source, composite super luminescent diode sources (cSLD) have been extensively used^43^ for HR OCT systems. At the time of writing, the 3 µm axial resolution threshold set here is within the claimed limits of current cSLD sources on the market. The costs of these are historically equal to or higher than lower cost SC sources and we are not aware of any cSLD sources that would go beyond 2.5 µm. In contrast, an SC OCT system's axial resolution is not limited by the light source, with resolutions engineered down to 1.5 µm in air well reported.^53^ The use of lower cost SC light sources in OCT systems is of current research interest,^54^^,^^55^ and in our previous work^19^^,^^29^ we have demonstrated that an LF SD format can be used to reduce the effect of their higher relative intensity noise (RIN). Here, we have demonstrated in vivo that this lower cost format is effective for imaging, producing clinical measures as accurate as any previous, more expensive, systems.

### Mirau Interferometer Discussion

In addition to the cost savings of an LF-SD approach to bring UHR OCT into the clinic, this work has also demonstrated the feasibility of including specific Mirau objective lenses in LF SD OCT systems (which has also recently attracted the attention of LF TD OCT system researchers).^27^ For this to work with low NA objectives, the slit separating the imaging spectrograph and interferometer parts in the design of the LiveOCT system is essential to confocally gate out directly reflected light from the Mirau beamsplitter. Low NA (high depth of field) objective options would be required for a system to be able to efficiently carry out standard (in this case, full corneal thickness) OCT imaging, therefore a slit is a practical requirement for the Mirau LF SD OCT systems.

Compact Mirau interferometric objectives reduce the required size for LF SD OCT systems and allow the possibility of clinical OCT systems with interchangeable objective lenses, akin to changing objectives on a microscope. If an application required higher lateral resolution but not high depth of field (e.g. cell counting), the operator could swap the objective of such a system to suit. The future development of single versatile (UHR-)OCT systems which could carry out the work currently undertaken by multiple different specialized instruments, such as current OCT, slit-lamp bio-microscopy, in vivo confocal microscopy, and specular microscopy (endothelial cell counting) by manufacturers is a possibility, providing clinicians with new options and opportunities when equipping their departments.

## Conclusion

With new hardware, measurement at the normal vector orientation, and segmentation over a reasonable length, a dataset was collected from nine healthy test subjects of central Bowman's and epithelial thickness. A repeatability of mean thickness of the Bowman's layer of 0.3 µm (1 standard deviation) for one test subject over a 55-day period, with different imaging operators. This repeatability matches the previous best reported value, with the common factors explaining this high repeatability being robust segmentation and imaging at near normal orientation to the interfaces. Note that the tear film and other supra-epithelium elements (contact lenses) do not significantly impact the expected repeatability of Bowman's layer thickness measurements. For the epithelial layer, the repeatability value for this study was 1.0 µm, the increase in comparison to Bowman's layer, also present in previous work, appears to be due to the variability of the tear film. From this work, we conclude the 95% population range for healthy adult in vivo central thickness for the Bowman's layer is 13.7 to 19.6 µm and for the epithelium is 41.9 to 61.8 µm.

Regarding the new hardware, to the best of the authors knowledge, LiveOCT is the first reported Mirau LF SD OCT system and first report of in vivo imaging with UHR LF SD OCT. The combination of LF and SC offers a cost-effective solution to get UHR OCT into routine clinical use. The combination with a Mirau interferometer design allows the future possibility of a subsequent device with interchangeable objectives, to give a broad range of lateral resolution properties to clinical users. The system has an axial resolution down to 2.4 µm in air (1.7 µm in corneal tissue, n_G_ = 1.387). The imaging speed was 204.8 K A-scans/second, but with the use of high-speed cameras MHz in vivo speeds are feasible with the technology.
